# Variability, Functions and Interactions of Plant Virus Movement Proteins: What Do We Know So Far?

**DOI:** 10.3390/microorganisms9040695

**Published:** 2021-03-27

**Authors:** Gaurav Kumar, Indranil Dasgupta

**Affiliations:** Department of Plant Molecular Biology, University of Delhi South Campus, New Delhi 110021, India; gauravkumar.bhu@gmail.com

**Keywords:** callose, coat protein, plasmodesmata, triple gene block, viral suppressor, virus movement, virus replication complex

## Abstract

Of the various proteins encoded by plant viruses, one of the most interesting is the movement protein (MP). MPs are unique to plant viruses and show surprising structural and functional variability while maintaining their core function, which is to facilitate the intercellular transport of viruses or viral nucleoprotein complexes. MPs interact with components of the intercellular channels, the plasmodesmata (PD), modifying their size exclusion limits and thus allowing larger particles, including virions, to pass through. The interaction of MPs with the components of PD, the formation of transport complexes and the recruitment of host cellular components have all revealed different facets of their functions. Multitasking is an inherent property of most viral proteins, and MPs are no exception. Some MPs carry out multitasking, which includes gene silencing suppression, viral replication and modulation of host protein turnover machinery. This review brings together the current knowledge on MPs, focusing on their structural variability, various functions and interactions with host proteins.

## 1. Introduction

**Figure 1:**
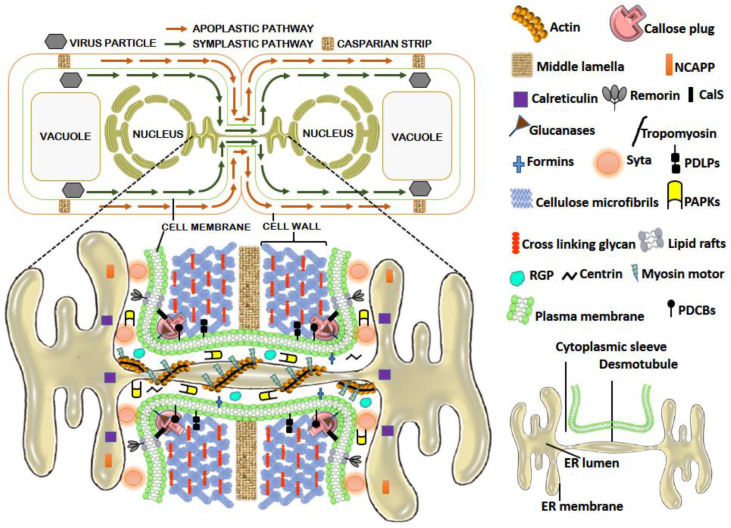
The symplastic and apoplastic pathways between adjacent plant cells and the model illustrating plasmodesmata (PD) structure with-associated cytoskeletal components and proteins governing PD permeability. Abbreviations: CalS—callose synthases; NCAPP—non-cell-autonomous pathway proteins; PAPK—plasmodesmal-associated protein kinase; PDCB—plasmodesmata-associated callose-binding proteins; PDLP—plasmodesmata-located proteins; RGP—reversibly glycosylated polypeptide; Syta—synaptotagmin A.

The process of infection of plants with viruses is broadly divided into two types; local and systemic. Local infection is often subliminal and is characterized by intracellular confinement of virus within and nearby the site of infection, while the systemic infection is progressive throughout the host and involves first, the short-distance movement of the virus from the infected cell to surrounding cells followed by long-distance movement using the host vasculature preferably the phloem tissue [1]. However, some phloem-limited viruses often skip the first route and are directly injected into the phloem by their vector interested in the phloem sap. In general, the plant viruses show a “symplastic” life cycle, i.e., from entry into the host cell to it accumulates in multiple copies; all occur in the cell symplast. The local intercellular virus spread in the host largely occurs through the symplastically connected cells via the plasmodesmata (PD, Figure 1) until they encounter the host vasculature for long-distance systemic infection [1,2].

The intercellular transport through PD depends upon its size exclusion limit (SEL), defined by the size of the largest molecule, which can pass through. Although solely dependent upon the hydrodynamic stokes radius [3], the PD SEL is highly dynamic and varies with cell-type, ambient light, temperature and developmental stages, being higher in some newly formed mesophyll cells compared to the fully differentiated mature cells [4]. Not surprisingly, the size of most viruses, (e.g., tobacco mosaic virus, TMV = 300 × 18 nm) or their nucleic acids, whether in the free or folded state, exceeds the SEL of most PDs (~60 nm) [5,6]. This indicates that the SEL of PD is modified during virus infection leading to systemic movement of viral progenies. Most of the plant viruses, if not all, encode a class of structurally diverse protein(s) known as movement protein (MP) that facilitates such intercellular adjustments for virus movements. These MPs have been reported to interact with various host factors to ensure successful virus movement across cells [5]. In general, the MPs perform the function of interacting with the viral genome, targeting them to PDs and modifying the SEL of PD, a process often termed as “gating”. The idea of synchronous coupling of virus replication and intercellular movement of viral genomes through MP-assisted PD-gating has further divided the movement into sub-stages involving post replication interactions, movement of an infection unit from the replication site to the gated PDs and the actual intercellular transport. This has further expanded the list of MP interacting partners [1,2,5,6].

Despite sharing a common function, there exists a large variation among MPs and their mechanisms of action. In recent years, a number of functions-associated with MPs, namely the mechanisms of modifying the PD SEL, formation of a viral transport complex, interaction with host components and suppression of RNA silencing, have been revealed. In addition, there are other viral proteins that mimic the functionality of MPs. In this review, we have chosen to bring together recent findings on MPs, interactions that they share and the division of labor that they show with other ancillary proteins required for the intercellular movement of plant viruses. Several other previous in-depth reviews present an exhaustive discussion on various aspects governing the intra-, and intercellular movement of viruses and the reader is guided to these for a more holistic understanding of this topic [7,8,9,10,11,12,13,14]. Additionally, we request readers to go through the reviews [5,15,16,17,18] to get an overview of the historical perspective and earlier findings, specifically on MPs and their interactions.

## 2. PD and PD Associated Proteins (PDAPs): The Facilitators of Virus Movement

PDs (Figure 1) are membrane-lined interconnected channels formed primarily upon entrapment of components of the endoplasmic reticulum (ER) in the course of phragmoplast formation during cytokinesis. PDs are centrally occupied by the appressed ER (desmotubule; DT), which brings about cytoplasmic continuity at the interface of adjacent cells. Surrounding the DT is a continuous channel of variable size called cytoplasmic sleeve, which is delimited by the plasma membrane (PM) [19] and is available for intercellular trafficking. Various PD-associated proteins (PDAPs; Figure 1) regulate PD permeability [20] and thus can affect the intercellular movement of viruses. The cytoskeleton proteins actin and myosin connect DT to PM through helically arranged globular particles and spoke-like tubular structures, thus, regulating the size of the cytoplasmic sleeve [21]. The SEL is negatively regulated by the deposition of callose (β-1,3-glucan). Callose is synthesized by callose synthase (CalS) and degraded by glucanases [22]. Calreticulin, a highly conserved Ca^2+^ sequestering chaperone protein, restricts the exit of misfolded proteins from the ER and accumulates in the PD [23]. The *Arabidopsis* calreticulin-1 (AtCRT1) shows a 22 amino acid (aa) PD-localizing signal sequence and accumulates around DT in PD, thereby blocking the movement of molecules across PD cytoplasmic sleeves [23,24]. Centrin is a Ca^2+^-binding contractile nano-filament protein localized in the PD-neck region, which negatively regulates the PD permeability upon dephosphorylation. Formin is an evolutionarily conserved integral membrane protein that regulates permeability by stabilizing and tethering the actin filaments to the PD membrane [25]. Lipid rafts are sterol- and sphingolipid-rich PM microdomains that regulate the callose homeostasis across PD [26]. Other PD-associated proteins affecting PD permeability include non-cell-autonomous pathway proteins (NCAPP) located near the orifice [27], PD-associated protein kinase (PAPK), carries out phosphorylation of NCAPP and other-associated proteins [9,27], PD-associated callose-binding proteins (PDCBs) abundant at PD neck region near the callose deposition site and affecting the callose metabolism [28], plasmodesmata-located proteins (PDLPs) promote callose deposition [29], lipid raft anchored protein (remorin) and reversibly glycosylated polypeptide (RGP) [30,31]. The *Arabidopsis* synaptotagmin A (SYTA) tethers the ER-PM contact sites across PD where it helps in endocytic recycling [32], tropomyosin binds to actin and regulates the myosin–actin binding, and myosin motor proteins act for the establishment of actin cables across PD and driving the cytoskeletal movements [33]. The locations of the above are depicted diagrammatically in Figure 1.

## 3. Types of MPs

With the pioneering study on temperature-sensitive mutants of TMV and the discovery of a virus-encoded nonstructural 30 kDa MP that assists the virus for its local spread [5,34], a large number of MPs have been discovered in several virus genera (Table 1). The criteria for a protein to qualify as MP is based upon: (a) loss of virus spread upon removal or mutation of the putative MP encoding gene fragment (b) use of transgenic plants expressing viral MPs or use of MPs of other related viruses for complementing viral movement where MP is removed or made non-functional (c) localization of MP-reporter gene fusions to the PD and (d) comparison of its sequence with a previously known viral MP.

Functionally, one or more than one viral proteins act as MP and facilitate virus intercellular movement by primarily influencing the host cellular systems for the regulation of PD permeability. On this basis, MPs can be divided into three major types: (a) PD-gating MPs that modify the PD SEL without any structural modifications in the PD (b) Tubule forming MPs that bring about structural modifications in PD by aggregating and forming “multisubunit tubular structures” that serve as conduits for the passage of virus particles and (c) MPs that facilitate virus movement across the cell by either/both gating the PDs and forming tubules. In addition, some of the viral proteins act as suppressors of RNA silencing (VSRs).

### 3.1. Viral Proteins (MPs) That Increase PD SEL without Any Structural Modifications in PD

The phenomenon of increasing the SEL of PD to allow macromolecular exchange, including the movement of virus particles, is termed as “gating”. Although the exact mechanism of gating still needs to be deciphered, to date, many models have been proposed. The PD-gating was initially observed in TMV MP-expressing transgenic plants, which showed a movement of much larger dextrans (9400 kDa) across PD compared to the SEL in control plants (700–800 kDa) [35]. In addition, the fluorescent dextrans were observed even far away from the primary microinjected site indicating that either the TMV MP itself moves across PD or initiates a series of diffusible signals that dilate PD at the distant site [122]. PD-gating may occur by a pore size increase caused by callose degradation upon action by β-1,3-glucanases (BGs). This causes an increase in the radius of the cytoplasmic sleeve, allowing virus movement [123]. There is also the possibility that the decrease in callose is attributed to downregulation or complete suppression of the callose synthase gene [124,125]. It is also proposed that the gating through MP may be due to its interaction with some PD-associated host proteins. An increased rate of TMV systemic spread and cell-to-cell movement was observed in tobacco plants with increased ankyrin ANK1 protein levels. ANK1 is normally a cytoplasmic protein, which upon virus infection is recruited to PD by the TMV MP. ANK, when coexpressed with TMV MP, caused reduction of callose deposits, thereby relaxing the callose sphincters at PD [36]. Among the PDLPs, which are known to be recruited by the tubule forming viruses (explained later), the PDLP5 initiates PD closure through callose deposition by stimulating callose synthase. The *pdlp5-1* mutants in tobacco reported an increase in cell-to-cell movement of TMV, although the actual interaction still needs further investigation [126]. In addition, the stress and damage to the cell wall upon virus entry causes methanol release from existing and newly synthesized pectin methylesterase (PME), which digests pectin and releases methanol vapors that activate the methanol-inducible genes (MIGs), including BG and NCAPP. BG promotes callose degradation, while NCAPP is a cellular factor that stimulates intracellular trafficking important for MP functioning [20,27]. New reports of PD lacking cytoplasmic sleeves [127] and lack of unambiguous electron microscopic evidence of PD dilation upon virus infection have made the existence of alternate options for intercellular movement of viruses a distinct possibility. Among the various virus genera, the intercellular movement through PD-gating is observed in viruses belonging to the genera *Dianthovirus*, *Carmovirus*, *Closterovirus*, *Luteovirus*, *Potyvirus*, *Tobamovirus*, *Tombusvirus* and some geminiviruses (e.g., Begomovirus). In addition, the PD-gating is also observed in genera *Benyvirus* and *Rhabdovirus* and in some members of the family *Alphaflexiviridae, Betaflexiviridae,* and *Virgaviridae* (Table 1).

#### 3.1.1. Characteristics of the TMV MP-The “30 K” Superfamily MPs

##### Tobamoviral MPs

**Figure 2:**
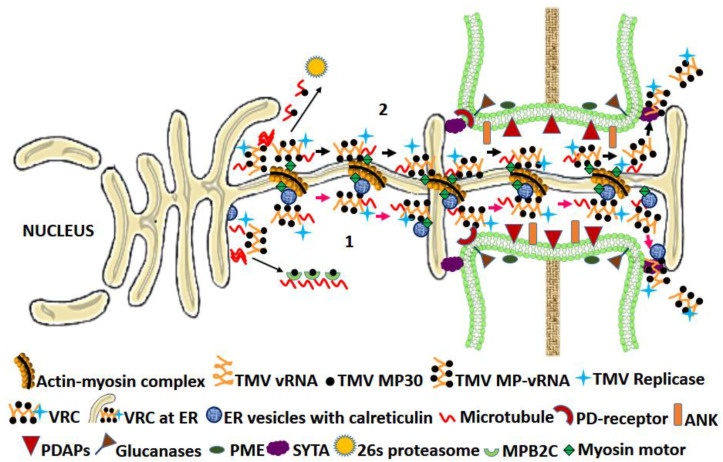
Model for intercellular movement of TMV: The TMV movement protein (MP) at the peripheral endoplasmic reticulum (ER) binds to viral RNA to form a vRNP complex (TMV MP-vRNA), which is joined by replicase to form a VRC. The VRC is delivered to PD either through calreticulin-containing ER-derived vesicle gliding through cell cytoskeleton constituted by the microtubule and ER-actin network (1; red arrows) [8,11] or under the influence of an MP-PLS [32,47,48], the VRC moves along the ER to PD, rafting over cytoskeleton driven by the myosin motor proteins (2; black arrows). Once the VRC reaches the PD, several PDAPs and other viral and host factors cumulatively work for PD “gating” [129]. Gating may occur by MP-mediated severing of actin microfilaments or by recruitment of specific β-1,3-glucanases for callose degradation. Additionally, the MP also interacts with the ANK host factor for downregulating callose. The cell wall-associated PME cause PD targeting of MP and assist gating [36,130]. The MP-PLS is recognized by SYTA, a tethering protein across ER-PM contact sites. These sites are recruited by MP for gating [32,49]. The microtubule near VRC may cause MP degradation via 26 s proteasome [6]. The MPB2C, a microtubule-associated plant factor, causes microtubular accumulation and binds to TMV-MP at the late infection stage to hinder its intercellular movement function [131]. Abbreviations: ANK—ankyrin repeat-containing; MPB2C—movement protein-binding 2C; PDAPs—PD-associated proteins; PLS—plasmodesmata localization signal; PME—pectin methyltransferase; SYTA—synaptotagmin A; VRC—viral replication complex; vRNP—viral ribonucleoprotein.

With around 37 species, the Tobamoviruses constitute the largest genus of the family *Virgaviridae*, having TMV as their type genus [128]. TMV requires a 30 kDa movement protein for their intercellular movement (Table 1; Figure 2).

Many other movement proteins have properties similar to that of TMV P30, and on the basis of this and their predicted secondary structure, they are grouped as the “30 K” superfamily [37]. Thus, a detailed study of the TMV MP generally serves as a blueprint for characterizing other similar MPs. Transmission electron microscopic studies revealed TMV MP to be localized in PD [38]. TMV MP binds to the vRNA in a sequence non-specific manner and facilitates its intercellular movement through PD in the form of ribonucleoprotein complexes (RNPs). In addition to PD, it also localizes to the peripheral ER membrane, where it forms viral replication centers (VRCs) [39,40]. The viral replicase acts synergistically with TMV MP, binds to RNP and facilitates its spread in PD desmotubule by lateral diffusion [41]. The presence of MP at ER cytosolic face, its association with the MT network for facilitating the VRC movement and the presence of the whole VRC-MP complex at the PD later was also demonstrated by fluorescence immunolabeling and electron microscopy [42]. The subcellular distribution and activation of TMV MP gating function is host-dependent and is regulated by C- terminal phosphorylation at Thr-256, Ser-257, Ser-261, and Ser-263 with either of the two host kinases, casein kinase 2 (CK2) and plasmodesmal-associated protein kinase (PAPK) sharing identical molecular weights. The regulation is, however, sequential, with initial activation of TMV MP gating function through phosphorylation at a single site, leading to successful virus intercellular movement followed by its inactivation by further phosphorylation events. This also provides a rationale behind the TMV infecting the plants (*N. tabacum*) even in the presence of the phosphorylation-based inactivation mechanism [43,44,45,46]. The plasmodesmal localization signal (PLS) in TMV MP resides in the 50-aa at the N-terminal end and is the first example of PLS in plant virus MP [47]. Other less efficient regions (from aa positions 61 to 80 and from 147 to 170) that functionally mimic PLS are also reported [47,48]. The MP PLS binds to the plant SYTA, which is localized to the plasma membrane, but more concentrated across PD where it tethers the ER-PM contact sites. These sites are then recruited by MP for remodeling the PD permeability [32,48,49,50]. This concept of MP-PLS to reach PD via SYTA interaction, however, does not support the ER-actin cytoskeletal involvement in the delivery of MP to PD since the PLS with its hydrophilic stretch of 50 aa lacks the features displayed by proteins interacting with the ER [40]. TMV RNP movement through ER membrane occurs by simple diffusion assisted by the host cytoskeletal elements. TMV spread depends upon myosin XI-2 [51]. TMV MP localization at PD was inhibited when the actin and ER membrane networks were disrupted. However, disruption of MT had no effect upon TMV intercellular movement, although, at permissive temperatures, TMV MP showed interaction with MT. In addition, the mutant plants with reduced MT dynamics were less susceptible to TMV [11]. These contradictory findings of PD localization either through MP-PLS or through ER-actin network indeed need further investigation. Moreover, there is still no direct evidence to show the interaction of MP with PD [11]. However, reports of indirect interaction of MP with callose through callose metabolism enzymes [52] and PDAPs for regulating the PD aperture [20] appear convincing. The TMV MP interacts with *A. thaliana* calreticulin-1 (AtCRT1) both in vivo and in vitro and co-resides with it at PD. Under stress and under overexpressed conditions, the increased calreticulin levels interfere with the TMV MP PD localization and instead directs it to the microtubular network away from PD [23]. As explained earlier, the PME initiates MIGs, including BG and NCAPP, that aid PD permeability and the intercellular movement of viruses. TMV MP interacts with PME in vitro, and deletion of domains responsible for this interaction hindered the ability of MP to facilitate the spread of viral infection [53]. Interestingly, plants expressing anti-sense PME showed delayed systemic spread of TMV [54].

In the case of TMV infection, it is observed that once the virus from the initially infected cell starts moving to the adjacent cells, the spread is even faster, and it seems that the MP initiates favorable conditioning of the surrounding cells before the cell is actually approached by the vRNA [55]. This prior conditioning event can be attributed to the early synthesis of TMV MP from the genomic vRNA and movement of TMV MP to the surrounding cells through PD-gating without even associating with the VRC. The former is possible due to the presence of an internal ribosome entry site that initiates prior translation of MP straightaway from the genomic RNA before even the synthesis of subgenomic RNA [56,57,58], while the latter can be explained by the interactome repertoire of MP, which includes the various PDAPs and other factors regulating the PD SEL [20]. The TMV-MP-mediated enhancement of systemic RNA silencing by facilitating the movement of siRNA across PD [59] also supports the function of TMV MP as an independent cellular conditioner for virus movement.

##### Dianthoviral MPs

The RNA2 component of red clover necrotic mosaic virus (RCNMV), a Dianthovirus encodes for a 35 kDa MP (P35, Table 1) that belongs to the 30 K superfamily, and like TMV, it does not require capsid protein for intercellular movement [60]. Localization studies of the GFP-fused MP mutants established a correlation between MP targeting to the cell wall and intercellular movement [61]. In addition, complementation studies of mutant MP with wild-type, alanine scanning mutagenesis and turnip crinkle virus-based GFP assay revealed definite domains for movement, complementarity and cell wall localization, PD-gating, cooperativity and RNA silencing suppression in RCNMV MP [62]. Using GFP-fused MP, Kaido et al. [63,64] showed colocalization of MP and replicase to the ER, for which C-terminal 70 aa of MP was crucial.

##### Geminiviral MPs

Among geminiviruses (Table 1), the DNA from the nuclei is shuttled to the cytoplasm via coat protein (CP; V1 ORF) in monopartite geminiviruses (e.g., Begomoviruses and Mastreviruses), while in bipartite, it is through the DNA B-encoded nuclear shuttle protein (NSP/BV1) [85,86]. Subsequent cytoplasmic localization to PD and further intercellular movement in monopartite geminiviruses is through an MP encoded by the V2 ORF or V3 ORF (e.g., beet curly top virus, a Curtovirus) or by the DNA-B encoded MP (BC1) in bipartite ones. Functional variation occurs in MP among monopartite viruses, for e.g., MPs in monopartite Begomoviruses has DNA-binding property, while MPs encoded by monopartite Mastreviruses do not bind to DNA [85,86]. Hence, it needs the viral CP to form a complex with the DNA, and the CP-DNA complex is then carried across PD by the MP. Such differences in the delivery of DNA from the nucleus to PD and across it are also found among bipartite Begomoviruses, for, e.g., abutilon mosaic virus (AbMV) shows “couple skating model” where NSP remains intact with the DNA from the nuclei and the NSP-DNA complex is then carried to PD and across it by the MP [132], while BDMV shows “relay race model” where the NSP hands over the viral DNA to MP from the nucleus into the cytoplasm, which is then carried to and across PD [133].

Similar to TMV, the MPs encoded by geminiviruses, squash leaf curl virus (SLCV) and cabbage leaf curl virus (CaLCuV) interact with *Arabidopsis* SYTA protein. Mutation in the *SYTA* gene hampered the intercellular movement of these viruses. It is proposed that the interactions of both MPs, encoded by CaLCuV and TMV with SYTA directs them to be loaded on early endosomes, which are then carried away by a recapture pathway to dock at the PD [134].

#### 3.1.2. The Triple Gene Block (TGB) Proteins

**Figure 3:**
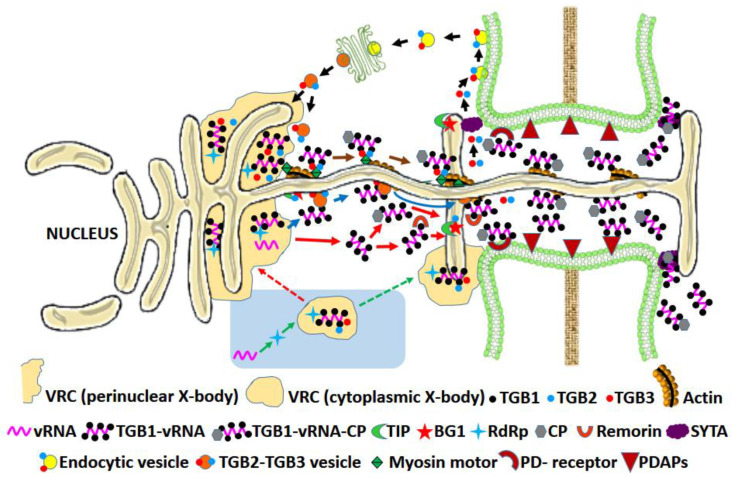
Model for TGB-mediated movement of PVX: The TGB1 organizes the ‘X-body’ (protective center of virus replication and assembly) and recruits TGB2 and TGB3 to it. TGB2 bridges the RdRp/dsRNA-TGBp3 interaction in the X-body. The vRNA in the X body replicates and forms VRC with ribosomes and viral RdRp [9]. The TGB1 alone or with CP binds to the vRNA at the VRC to form a vRNP complex (TGB1-vRNA/TGB1-vRNA-CP) that either directly reaches PD (orange arrows) or binds to the TGB2 and TGB3-associated vesicles and reaches PD along the ER through actin and myosin motor proteins guided by TGB3 (blue arrows) [8,17,129]. Alternatively, the VRC can be delivered to PD by TGB2 and TGB3 without vesicles (brown arrows). TGB2 facilitates the VRC fusion to PD. TGB1 and TGB2 perform PD-gating by interacting with remorin and β-1,3-glucanase-associated host factor TIP1, respectively, causing callose reduction. Subsequently, the vRNP complex is delivered to the adjacent cell leaving back TGB2 and TGB3 for recycling via endocytic pathway (black arrows) [18]. According to a recent alternative model (blue background) [93], the vRNA binds to RdRp in the cytoplasm forming the core replication unit later joined by TGB1, TGB2, TGB3 and CP to form a “cytoplasmic X-body”, which either joins the ER-associated perinuclear X-body (red dashed arrow) or directly reach the PD through TGB3 guided movement forming cap like complexes at PD (green dashed arrow). Abbreviations: BG1—β-1,3-glucanases; CP—coat protein; PDAPs—PD-associated proteins; RdRp—RNA-dependent RNA polymerase; TGB—triple gene block; TIP—TGB12K-interacting proteins; VRC—virus replication complex; vRNA—viral RNA; vRNP—viral ribonucleoprotein.

The triple gene block (TGB1, TGB2 and TGB3) is a specialized and evolutionary conserved group of nonstructural viral movement proteins found in 9 genera of plant viruses belonging to the families *Alphaflexiviridae*, *Benyviridae* and *Betaflexiviridae* (Table 1). Their structural features, interactions and role in the intercellular movement of the virus have been extensively reviewed ([12,18], and references therein). In addition to PD-gating and assisting the intercellular movement of plant viruses (Figure 3), the TGB proteins display a myriad of other functions in host cells [89]. The Potexvirus TGBp1 localizes partially in the nucleus and nucleolus, where it interacts with nucleolar proteins fibrillarin and coilin. These proteins may take part in the formation of viral cytoplasmic RNPs and thus take part in the long-distance movement of the viruses. The TGBp1 protein is also known to remodel the host actin and microtubular arrangement and putatively assist the virus movement-independent host protein trafficking to plasmodesmata [89]. The hordeiviral TGBp1 binds to host BSr1 R-protein and elicits hypersensitive response [90]. The potexviral TGBp1s have strong RNA interference suppression activities [91], a property described in detail later. The pepino mosaic virus (PepMV) TGBp1 interacts with host ROS scavenging enzyme catalase 1, resulting in the weakening of the ROS-mediated plant defense mechanism [92]. The potato virus X (PVX) TGBp2 plays a role as the molecular adaptor in viral replication by interacting with the C-terminal domain of RdRp and forming chain-mail-like aggregates around RdRp that further localizes TGBp3 aggregates to enhance viral replication [93]. The PVX TGBp3 is responsible for virus-induced unfolded protein response under ER stress and upregulates the ER-resident and ubiquitin ligase chaperones [89].

#### 3.1.3. Potyviral MPs

**Figure 4:**
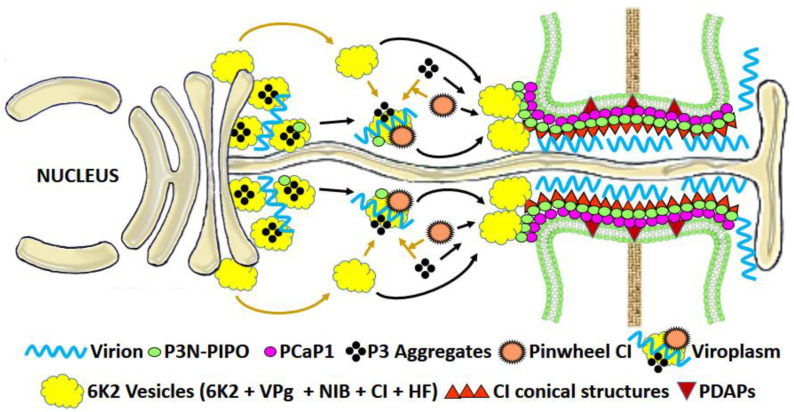
Model for movement of TuMV: The TuMV virion movement involves a multifaceted interaction between virus-encoded proteins 6 K2, VpG, NIb, CI and P3N-PIPO and host-encoded PM localized Ca^2+^-binding protein PCaP1. The TuMV MP P3N-PIPO interacts with PCaP1 for its localization to PD. After translation, the virus-encoded proteins for replication along with the assisting host proteins get assembled into clusters of 6 K2 vesicles. The ER-derived P3 aggregates interact with 6 K2 via the P3C domain and accumulate in the 6 K2 vesicle cluster. Along with PD, the P3N-PIPO also localizes to the 6 K2 clusters via P3. The CI also gets recruited to two sites- the PD, by interaction with P3N-PIPO-associated with PCaP1 at PD and-6 K2 clusters by interaction with P3-colocalized P3N-PIPO where probably they participate in replication. The PD recruited CI forms a CI-P3N-PIPO complex to which more CI molecules join via self-interaction resulting in the formation of conical structures. The CI also self-integrates to form a cytosolic pinwheel-like structure. The CK2 vesicle cluster either combines with CI, P3 and P3N-PIPO to form a virus-induced cytosolic viroplasm or is directly delivered to PD and docked at the P3N-PIPO-associated conical CI structures. Upon reaching the PD, the viral RNA is encapsidated by CP to form an intact virion or RNP complex, which is delivered to the neighboring cell by the CI. Adapted and modified from [80]. Abbreviations: CI—cylindrical inclusion; HF—host factor; NIb—nuclear inclusion protein; PDAPs—PD-associated proteins; PM—plasma membrane; RdRp—RNA-dependent RNA polymerase; SYTA—synaptotagmin A; TuMV—turnip mosaic virus; VpG—viral protein genome-linked.

PD-gating for intercellular movement is also observed in members of genus *Potyvirus*, the largest group of flexible filamentous viruses, where virus-encoded proteins destined for different functions helps in virus cell-to-cell movement. The PD located protein P3N-PIPO is a dedicated MP in the Potyvirus, turnip mosaic virus (TuMV) [76]; it directs the viral cylindrical inclusion protein (CI) to form PD-associated conical structures that assist in intercellular movement (Figure 4).

It has been recently shown that the CP with its vRNA interacting conserved core and C-terminal domain also participates in intercellular trafficking of the virion [77]. The well-known potyviral RNA silencing suppressor HC-Pro helps in Potyvirus movement by increasing the PD SEL in coordination with CP [78]. TuMV P3N–PIPO recruits the PM-associated Ca^2+^-binding protein 1 (PCaP1) to PD. PCaP1 can sever actin filaments, which is required for the intercellular movement of the virus [79]. Notably, both P3N and PIPO domains of TuMV P3N–PIPO are essential for intercellular movement. The PIPO domain is important for its interaction with CI, while the P3N domain for its interaction with P3. The Potyvirus 6 kDa (6 K2) membrane protein interacts with the host ER for the biogenesis of cytoplasmic membranous vesicles, the site for virus replication. The shared N terminus of P3N-PIPO interacts with P3, which recruits the 6 K2-induced vesicles to the PD, where they are docked at the CI-induced conical structures that assist in intercellular movement [80].

### 3.2. Viral Proteins (MPs) That Increase PD SEL by Structural Modifications Caused by Tubular Aggregates

The intercellular movement in some viruses involves intact virions to be moved across the cell [9]. These viruses modify the normal PD architecture for their intercellular movement by forming specialized structures (tubules) across cells by the oligomerization of their MP and, sometimes, CP [135]. In the transiently transfected protoplasts and/or insect cells, the MPs of these viruses undergo oligomerization and form tubular structures that protrude out from the plasma membrane indicating that in general, the MP in itself is capable of tubule formation. Neither CP nor any other host cellular structure (e.g., PDs) is needed for the formation of tubules [96,108,109,136]. The tubule formation for intercellular movement is found in viruses belonging to the genera *Caulimovirus*, *Tospovirus*, *Umbravirus* and members of subfamily *Comovirinae* of the family *Secoviridae* (Table 1).

#### 3.2.1. Caulimoviral MP

**Figure 5:**
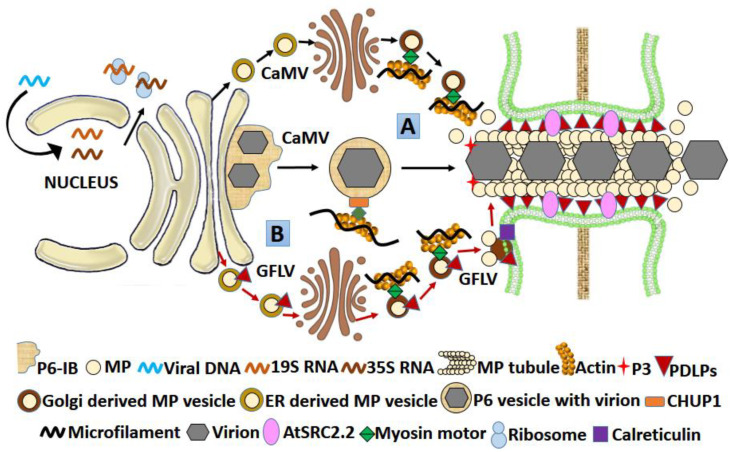
Model for tubule formation by MP in CaMV and GFLV (A) The CaMV vDNA transcribes in the nucleus to 19S and 35S RNA, which translate in the cytoplasm. The 19S RNA encodes a 58 kDa P6 protein that forms ribosomes rich IB (virus factory), the center for other virus proteins translation (e.g., P1-MP, P3, P4-CP, etc.) by 35S RNA. The MP reaches PD through vesicular transport via secretory pathway (black arrows), resulting in multiple MP copies that form tubule across PD. Once the virus attains a threshold copy number in IB, the P6 protein detaches as a vesicle and is assisted by CHUP1 to move over the actin filament network. After the virion reaches PD, the MP interacts with the virion (vDNA+56 kDa CP) through P3. PDLPs (also an MP receptor) and AtSRC2.2 at the PD help in the virion delivery. The virion delivery to the next cell putatively occurs through a tread-milling mechanism where there is unidirectional addition of vDNA-P3-CP-MP subunits assembly at one end and subsequent disassembly at the other, causing the virion delivery [7,137]. (B) The virion delivery is similar in GFLV, but here the MP first reaches the calreticulin-rich sites on the PM and thereafter, it reaches the PD by diffusion (red arrows). The PDLP is also delivered to PD by diffusion through PM, where it reaches via a secretory pathway in association with class XI, XI-K and XI-2 myosin [11,129]. Abbreviations: AtSRC2.2—*Arabidopsis thaliana* soybean response to cold; CaMV—cauliflower mosaic virus; CHUP1—chloroplast unusual positioning protein; GFLV—grapevine fanleaf Nepovirus IB—inclusion bodies; PDLP—plasmodesmata localized protein; vDNA—viral DNA.

In Caulimoviruses, the most studied movement protein is that of its type species cauliflower mosaic virus (CaMV). Its tubule forming RNA-binding MP is a 38 kDa protein encoded by the ORF1 (P1; Table 1; Figure 5) [96,97]. The tubule-forming capacity of CaMV MP was also demonstrated in infected protoplasts and insect cells [96]. CaMV MP interacts with host PDLPs, the MP receptors at PD [98], but the actual mechanism of tubule formation involving PD desmotubule replacement, increase in SEL and oligomerization of MP to form tubule is still unclear. Intact virions traversing the PD through a tubular structure have been clearly revealed by electron microscopy [99].

#### 3.2.2. Tospoviral MP

In the tomato spotted wilt virus (TSWV), a Tospovirus, the NS_M_ protein is the MP as displayed by its properties of oligomerization and formation of tubular structures in infected protoplasts and insect cells. It forms and extends the tubule across PDs, assisting the intercellular movement of the non-enveloped nucleocapsids [108]. In the thrips vector *Frankliniella occidentalis*, TSWV NS_M_ protein is functionally linked with autophagic pathways, not involved in assisting the intercellular movements [110].

#### 3.2.3. Umbraviral MP

The ORF-4 of the groundnut rosette virus (GRV), an Umbravirus, encodes for 28 kDa protein that is PD-localized, binds to both ssDNA and ssRNA cooperatively in a sequence nonspecific manner and induces tubule formation on the protoplasts surfaces in *N*. *benthamiana* [111,112]. Umbraviruses do not encode for their own CP and require a helper Luteoviridae member for virion formation and vector transmission. This reduces MP dependency on CP, and as indicated by the protoplast experiments, among Umbravirus members, MP in itself is self-sufficient for tubule formation and intercellular movement of virion [138].

#### 3.2.4. MPs of Comoviruses, Fabaviruses, and Nepoviruses

As indicated by the mutagenesis studies, the tubule forming MPs in members of sub-family *Comovirinae,* Comoviruses, Fabaviruses, and Nepoviruses have molecular weights of 48/58 kDa, 37 kDa and 38–43 kDa, respectively (Table 1) and are products of polyproteins encoded through their RNA-2 [96]. Tubule formation by the MP of cowpea mosaic virus (CPMV), a Comovirus, was demonstrated in both infected protoplasts/insect cells and infected plant material where clear tubular structures containing virus particles superseding the PD desmotubule were observed under an electron microscope. Studies using antisera and mutagenesis confirmed the 48 kDa moiety of the 58 K/48 K protein to be crucial for tubule formation [136,139]. Tubule formation was also observed in grapevine fanleaf Nepovirus (GFLV; Figure 5) and broad bean wilt virus 2 (BBWV-2) a Fabavirus [100,101,109]. The MPs of these viruses have different binding affinities for CP. Both CPMV and BBWV-2 encode a large CP of 35–40 kDa and a small CP of 20–25 kDa, but the MP of the former binds to the large CP, whereas in the latter, it binds to the small CP [140,141]. The C-terminal domain of MP was found to be crucial for this interaction; a CPMV MP with C-terminal deletion showed an empty tubule with no virions [103], a similar deletion in GFLV MP abolished the interaction with CP and restricted the systemic spread [102]. However, whether the C-terminal deletion abolished the interaction of CPMV MP with the CP is not clear. Combining both, it was postulated that in both CPMV and GFLV, the C terminal domain of MP is crucial for association with CP and virion formation so that an intact virion moves intercellularly via tubules. The tubule formation was later-associated with the N-terminal and central region of the CPMV MP, while the virion incorporation in the tubules was defined as a function of its C-terminal domain [104,105]. Deletion and point mutation studies in CPMV MP showed that initially, the MP is targeted to plasma membrane involving its oligomerization (involving aa 228–251); later, it accumulates in spots after possible interaction with some host protein (involving aa 252–276). Finally, tubules assemble (involving aa 293–298 of MP) from the spots, which traverse the PD, replacing the desmotubules. The process culminates with the delivery of the virion in an adjacent uninfected cell and disassembly of the tubule [106,107]. The host PDLPs have no role in the transfer of viral particles, but they may act as PD receptors for MP, assisting them in oligomerization to form tubules [98].

### 3.3. MPs That Increase PD SEL with or without Any Structural Modifications across PD

Apart from the exclusive nature of either gating or tubule forming, members of the family Bromoviridae harbor certain MPs that apparently perform both gating as well as tubule forming functions (Table 1). The MPs of viruses belonging to genera *Alfamovirus*, *Bromovirus*, *Cucumovirus,* and *Ilarvirus* of family *Bromoviridae* increase the PD-SEL, are PD-localized, bind to RNA and show molecular weights between 32 and 36 kDa. They are encoded by the 3a gene fragment of RNA3 [117,142].

Among these members, a significant variation is observed between species and strains regarding the requirement of CP for the movement-related functions of MPs [113,114,143].

The intercellular movement of the alfalfa mosaic virus (AMV) requires both MP and CP. The MP induces tubule formation and exhibits RNA-binding capacity in a sequence nonspecific manner. An intact virion formation is not necessary for the intercellular movement of the virus; however, the association of MP with CP is crucial, as observed by restricted intercellular movement following mutation of CP [113].

While most of the brome mosaic virus (BMV) strains do not require CP for intercellular movement [115], the tubule forming M1 strain [114] requires CP in addition to its MP when its intercellular movement was tracked in *Chenopodium* sp. The MP interacts with *N*. *benthamiana* protein (NbNACa1), which apparently regulates its localization on PD [116].

In another Bromovirus, the cowpea chlorotic mottle virus (CCMV), MP is sufficient (no CP required) for intercellular movement. Interestingly, the rate of intercellular movement is host-dependent, being faster in an experimental host (*N*. *benthamiana*) than its natural host (cowpea), as demonstrated when CP was replaced by an enhanced GFP [144]. When the MP gene of BMV and CCMV were exchanged, and the CP expression was impaired, the recombinant CCMV harboring BMV MP showed restricted movement, while no effect on intercellular movement was observed in the case of recombinant BMV harboring CCMV MP. This qualifies MP as the chief determinant of the virus-specific CP functions for intercellular movement in Bromovirus [145]. However, considering the above facts, the CP and MP interaction and dependency need to be further examined in light of the differential response observed between virus strains and species as well as their host.

The cucumber mosaic virus (CMV) tubule forming MP is PD-localized and binds to ssRNA [118]. CMV MP requires CP assistance for intercellular movement. However, the CP-dependency was abolished when a truncated MP was used, having 33 aa removed from C-terminal [119]. The truncated MP, as observed through atomic force microscopy, showed a strong binding affinity for viral RNA and formed a more condensed RNP complex compared to a native MP requiring CP [120]. The CMV MP showed tubule formation on the protoplast surface; however, no such tubules were observed in CMV-infected *N*. *clevelandii* as observed by quantitative immunogold labeling of the MP of CMV. However, the MP was observed at both the entry and the central cavity of the PD pore, as well as the distant connecting sieve elements. In addition, CMV having a mutation in the 3a gene encoding the MP was still able to infect tobacco both locally and systemically, although protoplasts containing such mutants showed no tubule formation [117].

The MP of prunus necrotic ringspot virus (PNRSV), an Ilarvirus, also binds to ss RNA in a sequence-nonspecific manner. A 33 aa domain at the N-terminal of MP is crucial for ss RNA binding as found out by deletion mutagenesis followed by Northwestern analysis. The N-terminal location of the RNA-binding domain in MPs encoded by Ilarviruses and Alfamoviruses are different from MPs encoded by other members of the family *Bromoviridae* where the similar domain lies at the C-terminal. This may have evolutionary implications showing phylogenetic divergence among viruses belonging to the family *Bromoviridae* [121].

Members of the family *Bromoviridae* also show high functional variability in the dependence of their MPs on CP as well as tubule formation. With the current findings, a correlation between CP interaction and tubule formation by the MP can be established. MPs requiring CP for virus movement may form tubule, and the ones, which do not require CP may move by gating the PD. However, it requires further conclusive studies involving both the variability in virus species and strains as well as the host range to establish this functional relationship.

### 3.4. MPs as RNA Silencing Suppressors

**Figure 6:**
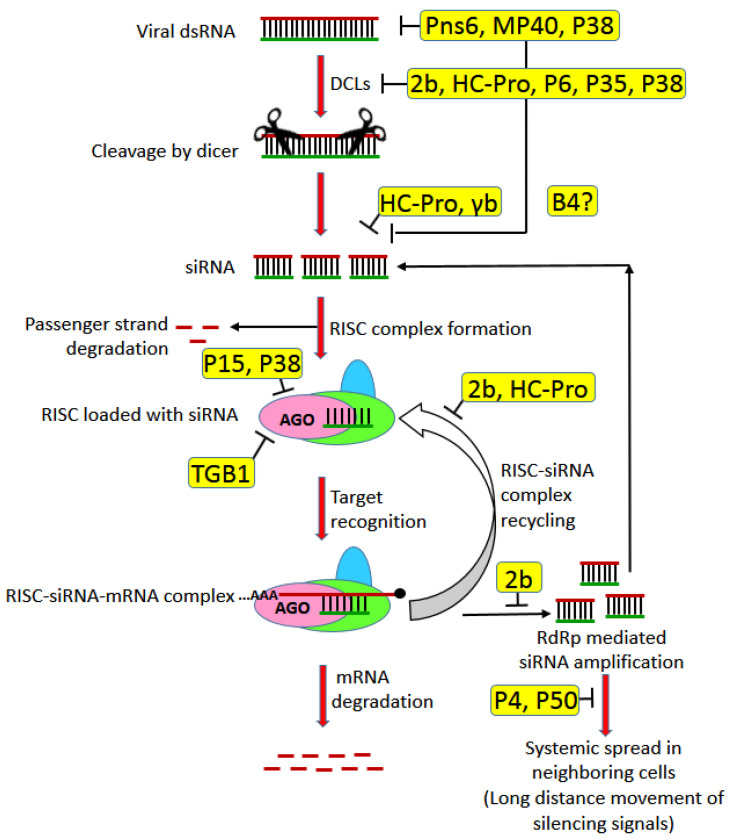
Schematic illustration of the action points of the MPs or movement assisting proteins acting as VSRs over basic RNAi mechanism layout: The PVX TGB1 targets AGO1 and causes its degradation through the proteasome pathway [150]. The RRSV Pns6 targets the upstream step of viral dsRNA formation [151]. The ACLSV P50 is a suppressor of systemic silencing by inhibiting the systemic movement of silencing signals [152]. The CLBV MP40 acts as a local silencing suppressor by putatively affecting the dSRNA and siRNA generation [153]. The P4 movement protein in Luteovirus has been recently identified as a systemic RNA silencing suppressor [84]. The P6 protein of CaMV acts as a silencing suppressor by indirectly blocking the DCL4 [154]. The 2b protein of CMV act as a silencing suppressor by interacting with DCL1, AGO1 and 4, siRNA biogenesis and RdRp downregulation [155]. The Potyvirus HC-Pro selectively binds to siRNA of different sizes, blocks HEN1 methyltransferase, binds and inhibits HEN1, prevents AGO1 loading, downregulates AGO1 by upregulating its corresponding micro RNA and may be involved in AGO3 cleavage, interact with RAV2 factor, thus blocking the siRNA biogenesis [155]. The BSMV γb binds to siRNA [156]. The PCV P15 interacts with AGO1 and prevents the siRNA binding [156]. The RCNMV P35 suppresses RNAi probably by sequestering DCL1 and using its helicase activity for its own replication [62]. The P38 CP, which assists TCV movement, is an RNAi suppressor, which binds to AGO 1 and 2, upregulating the DCL1 for antagonizing the functions of DCL3 and 4, binding to dsRNA and preventing primary siRNA biogenesis and upregulating AGO1 specific miRNAs [155]. The B4 protein of BBTV is a silencing suppressor; however, the exact target for suppression is not found yet [157]. Abbreviations: VSRs—viral suppressors of RNA silencing; PVX—potato virus X—RRSV—rice ragged stunt virus; ACLSV—apple chlorotic leafspot virus; CLBV—citrus leaf blotch virus; CaMV—cauliflower mosaic virus; CMV—cucumber mosaic virus; DCL—dicer-like; AGO—Argonaute; siRNA—small interfering RNA; RdRp—RNA-dependent RNA polymerase; HC-Pro—helper component proteinase; HEN1—HUA enhancer 1 (small RNA methyltransferase); BSMV—barley stripe mosaic virus; PCV—peanut clump virus; RCNMV—red clover necrotic mosaic virus; TCV—turnip crinkle virus; CP—coat protein; BBTV—banana bunchy top virus.

RNA interference (RNAi) is a fundamental mechanism of regulation of gene expression in eukaryotes both at the transcriptional and post-transcriptional level by specific mRNA degradation through complementary small RNA [146]. RNAi is used by plants as an important anti-viral defense through degradation of the viral RNA [147]. Some viruses mount a counter-defense at the sites of VRC from RNAi by compartmentalization using subcellular structures, e.g., ER spherules or by speeding up the replication and systemic infection process outpacing the mobile RNAi signals [148]. Most viruses, however, mount counter-defense by expressing proteins called viral suppressors of RNA silencing (VSRs) [147]. The VSRs are generally multifunctional in nature, and some of them, in addition to the inhibition of specific steps of the RNAi pathway, is also involved in virus replication and movement. Interestingly, a large number of MPs have also been shown to have VSR activities (Table 1). Although there is a report that the TMV MP controls its own propagation via promoting the spread of host RNA silencing [149], most of the other viral MPs act as VSR and suppress the host defense system (Table 1; Figure 6).

In this regard, it is important to note that either the MP in itself or another movement-associated protein may act as VSR (e.g., Luteoviral P4 or Potyviral HC-Pro) [84,158] or the replicase-type proteins involved in the formation of transport complexes, such as VRCs (e.g., 126 kDa TMV replicase) [159]. The synchronicity of transfer of VSRs with the viral genome also plays a critical role in preventing the viral genome from degradation through RNAi and thus promotes pathogenicity. If the MPs having VSR activity get transferred to the new cell first and then the viral genome enters it through plasmodesmata, the former can condition the new cell for the proper proliferation of the latter. If the MPs are a part of VRCs (e.g., 126 kDa TMV replicase), the VSR activity should go hand-in-hand with the viral infection [130,160,161].

## 4. Conclusions

It is abundantly clear that MPs have evolved diverse mechanisms to accomplish their goal of ensuring the transport of viruses across the host imposed natural barriers (e.g., rigid cell walls) and provoke a successful, productive systemic infection. Although highly variable, MPs interact with a plethora of viral as well as host factors, facilitating the movement of the viral genomes to and through PD. Studies on viral MPs indicate that the virus movement is spatially and temporally coupled with replication, encapsidation and suppression of host RNAi-mediated defense, and all this involves a close association of viral MPs with the host PD, cell cytoskeleton, endomembrane system and the secretory pathway.

Further progress in our understanding of their actions will probably be achieved by revealing finer details of their structures and interactions with components of PD and other viral proteins. Whether MPs also play a role in the tissue tropism of certain viruses may be revealed by better understanding the functions of various PD types in tissues and their interactions with MP. A new aspect of the function of MPs is emerging when one considers their role in non-cell-autonomous functions in plants, a subject which may have implications in viral pathogenicity. Several outstanding questions that can be put forward are:

What is the role of the tertiary structure of MPs on their interactions with key proteins of PD? How does the specific function of MP change upon post-translational modifications? Are there variants of PD-localization signal in MPs structurally different from each other? If yes, then how do the various membrane contact sites/receptors differentiate between these signals? Is it possible that the limited non-cell-autonomous functions known in plants are intimately modified by MPs? Is cellular conditioning at the infection front before the virus spread a general phenomenon acquired by viruses of the “30 K” superfamily clan, or the mechanism extends to other viruses with non-related MPs too? How does the role of MPs known in RNA silencing suppression impact their effect on the intercellular spread of viruses? In tubule-forming viruses, what is the mechanism by which MP modifies the PD components in forming the tubules?

Finally, one can look forward to further developments in super-resolution- and fluorescent probe-based 3-D microscopic techniques for a clearer picture of the interactome of MPs. In addition, the detailed study on MP interaction and function can yield a significant amount of information about the response of the host cellular machinery, not only towards viral infection but also may provide a novel handle for the better understanding of cellular machinery for intercellular movement of macromolecules and other substances (both cellular and foreign) across PD.

## Figures and Tables

**Table 1:** General details of movement proteins of some of the most worked out viruses across genera.

Family/Genus	Species	MP	MP—Properties	MP—Mode of Virus Intercellular Movement	Assisting Viral Proteins and Their Putative Function	Interacting Host Proteins and Their Putative Function	References
*Virgaviridae/Tobamovirus*	TMV	30 kDa (P30)	α-Helical domain rich Binds ssRNA PD-localized Membrane-bound	PD-gating (increases SEL) Helps in the formation of replication complex and, along with p126 (silencing suppressor), participates in intracellular transport	Replicase—binds to vRNP and along with facilitates its movement across PD	PME—cell wall receptor, PD delivery PAPK, CK2, RIO kinase—MP phosphorylation KELP, MBF1—transcriptional co-activators MPB2C—subcellular localization by the microtubular association of MP Actin—movement of vRNA along ER increase PD SEL EB1a—microtubular association of MP for vRNP movement ANK—cytoplasmic receptor for MP Tubulin and γ-tubulin—movement of vRNA NtMPIP1—DnaJ-like chaperone assisting movement Calreticulin—movement of the viral ribonucleoprotein SYTA—recognizes the MP PLS, remodeling of the PD permeability	[1,11,15,22,35,36,37,38,39,40,41,42,43,44,45,46,47,48,49,50,51,52,53,54,55,56,57,58,59]
*Tombusviridae/Dianthovirus*	RCNMV	35 kDa (P35)	Binds ssRNA Have localization domains for both cell wall and ER Silencing suppressor Host range determinant	PD-gating (increases SEL)	Viral replicase complexes formed with RNA1—recruits MP to punctate cortical structures of ER, which is essential for intercellular movement MP—interacts with CP for long-distance systemic movement	NbGAPDH—A intercalates between VRC and MP and facilitates intercellular movement	[60,61,62,63,64]
*Tombusviridae/Carmovirus* MPs-(often termed double gene block proteins)	TCV	P8 and P9	P8—binds ssRNA P8—nuclear-localized P9—cytosolic and ER membrane-localized	PD-gating (increase SEL)	CP—only for long-distance systemic movement through an assemblage of the virus particle and supportive silencing suppression activity	P8—interacts with Atp8 with two “RGD” sequences-cytoskeleton trafficking interactions for virus movement	[65,66]
	CarMV	P7 and P9	P7—binds ssRNA P9—no RNA-binding activity P7—cytosolic initially, later localized near the cell wall P9—probably localized to ER membranes	PD-gating (increase SEL)	NK	NK	[67]
	PFBV	P7 and P12	P12—binds RNA P12—localized to ER membranes	PD-gating (increase SEL)	NK	NK	[68]
	MNSV	P7A and P7B	P7A—binds RNA P7B—no RNA-binding activity P7B—probably localized to ER membranes, silencing suppression activity	PD-gating (increase SEL) P7B accumulates on Golgi and modulates actin filaments to PD	CP—R2-subdomain, also a VSR	Movement is energy-dependent on unknown host protein (s)	[69,70]
*Kitaviridae/Higrivirus*	HGSV	BMB1 and BMB2	BMB1-binds RNA BMB2—no RNA-binding activity BMB2—an integral ER protein, induces ER constriction, acquire W-like topology like reticulons and also forms a PD-associated replication compartment BMB2—can mediate the transport of BMB1 to and through plasmodesmata	PD-gating (increase SEL)	NK	NK	[71,72]
*Tombusviridae/Tombusvirus*	TBSV	P22	Binds RNA Have regulatory sequences for RNA accumulation Induces HR-like necrotic local lesions on *Nicotiana edwardsonii*	PD-gating (increases SEL)	P19—assist systemic movement through silencing suppression	HFI22—leucine zipper homeodomain protein interacts with P22 for delivery of P22/RNA complexes through PD for intercellular movement	[73]
*Closteroviridae/Closterovirus*	BYV CTV	P6, P64, CP, CPm and HSP70h	P6—RER-associated PD-localized (HSP70h) MT-binding (HSP70h) Virion assembly (CP, CPm, and HSP70h)	PD-gating (increase SEL)	P20—interacts with HSP70h for long-distance systemic movement	Class VII myosins—motility and targeting of HSP70h to PD	[74]
*Closteroviridae/Crinivirus*	LIYV	P6, P64, CP, CPm and HSP70h P26 (essential for virus systemic infection)	PD-localized Forms conical PM deposits (PLDs) at PM over PD pit fields	PD-gating (increases SEL)	P9—unknown function	NK	[75]
*Potyviridae/Potyvirus*	TEV, TuMV, SMV, BCNMV, LMV	P3N-PIPO, CP (TEV/TuMV/SMV) CP and HC-Pro (BCNMV/LMV)	CP—N terminal and central core domain participates in the movement HC—Pro-silencing suppressor P3N-PIPO—localized to PM and PD	PD-gating (increase SEL)	CI—directed to PD by P3N-PIPO and forms conical structure aiding intercellular movement CP— (*cis* expressed) associates the RNP complexes (RNPs) P3—recruits a small portion of P3N-PIPO to the 6 K2 aggregates	pCAP1—binds to P3N-PIPO aids its localization to the PD and intercellular movement	[76,77,78,79,80]
*Luteoviridae/Polerovirus*	PLRV, TuYV, PeVYV	17 kDa (P17) 175 kDa) P4 (PeVYV)	Binds RNA PD-localized Phosphorylated (host-dependent)	PD-gating (increase SEL)	P3a—localization to the outer membrane of mitochondria and plastid	PKC—related membrane-associated protein kinase phosphorylation Actin—intracellular trafficking and PD localization	[81,82,83]
*Luteoviridae/Luteovirus*	BYDV	17 kDa (P4)	Binds RNA PD-localized Silencing suppressor Cause PCD Nuclear membrane targeting Self -interactive	PD-gating (increases SEL)	P3a—localization to the outer membrane of mitochondria and plastid	NK	[84]
*Geminiviridae/Begomovirus*	BDMV AbMV, ToLCNDV	BC1 (BL1)	Binds ss/ds DNA PM, cell wall and nucleus localized Influence the symptom severity Phosphorylated ToLCNV (BC1 MP) is a determinant of mechanical transmissibility	PD-gating (increases SEL)	BV1-NSP—replicated viral genome delivery from the nucleus to the cytoplasm Formation of a nucleoprotein complex CP—for long-distance movement	PAPK—MP phosphorylation Histone H3—formation of DNA-H3-NSP-MP complex in BDMV Hsp-70—assist AbMV movement through stromules and plastids SCD-2—AbMV movement	[85,86]
*Rhabdoviridae/Nucleorhadbdovirus*	RYSV, SYNV, MMV, MFSV, PYDV, EMDV, CaLCuV	P3 (RYSV and MMV) P3 or sc4 (SYNV) P4 (MFSV) Y (PYDV and EMDV)	Secondary structure similarity with 30 K superfamily PD-targeted P3 and sc4 binds RNA nonspecifically sc4 is membrane-associated	PD-gating (increase SEL)	M (matrix) and G (glyco) protein—formation of movement complex (PYDV) G (glyco) protein— formation of movement complex (SYNV)	P3 or sc4 (SYNV) —interacts with MT localized sc4i21 and sc4i17 (homologs of the *Arabidopsis* phloem-associated transcription activator AtVOZ1) —for intracellular trafficking and aiding in the intercellular movement	[87,88]
*Rhabdoviridae/Cytorhabdovirus*	LNYV, ADV, TYMaV	4b (LNVY) P3 (ADV and TYMaV)	P3—PD-localized 4b shows nuclear localization Membrane-associated (4b and P3)	PD-gating (increase SEL)	P (phospho) protein— formation of movement complex (ADV)	P3 (LNVY) —interacts with MT-associated VOZ1-like transcriptional activator—for anchoring the virus movement complexes to the MT network for intracellular trafficking and aiding in the intercellular movement	[87,88]
*Alphaflexiviridae/Potexvirus* and *Betaflexiviridae/Foveavirus*^a^	PVX GRSPaV	TGB1 TGB2 TGB3 TGB1 TGB2 TGB3 Some members have TMV 30 K-like MP	TGB1—25 kDa binds to ssRNA, ATPase/helicase activity, RNA silencing suppressor, translation activator, organization of VRC (X-bodies) through ER/actin remodeling TGB2—12 kDa, ER transmembrane protein TGB3—8 kDa, ER transmembrane protein, induces PCD in *N. benthamiana*	PD-gating (increase SEL) TGB2—provides the environment for robust virus replication	TGB1—CP TGB2—viral RdRp	TGB1- Actin—organization of PVX-X bodies Remorin—interaction impairs the virus movement Fibrillarin and coilin—assist in vRNPs formation CK2-like kinase—for MP phosphorylation TGB2- TIP—interacts with BG1 for regulating callose accumulation NbCPIP2a and NbCPIP2b—interacts with PVX RNA and CP for replication and movement	[12,18,37,89,90,91,92,93,94]
*Virgaviridae/Hordeivirus*	BSMV	TGB1 TGB2 TGB3	TGB1—42–63 kDa, have nucleolar and nuclear localization signals, binds to RNA, NTPase/helicase activity causing unwinding of virus RNA duplex TGB2—13–14 kDa ER transmembrane protein TGB3—17 kDa ER transmembrane protein, PD targeting	PD-gating (Increase SEL) TGB3 interacts with TGB1 and TGB2 and provides a basic framework for RNP formation	TGB1—interacts with CP TGB2—replicase γb (also a VSR)- enhances the ATPase-mediated assembly of BSMV-RNP complex	Fibrillarin—formation of RNP for intercellular BSMV movement CK2—protein kinase, phosphorylates TGB1 for intercellular BSMV movement Actin—intercellular BSMV movement and TGB3 localization to the cell wall	[18,89,90,91,92,93]
*Bunyaviridae/Benyviruses*	BNYVV	TGB1 TGB2 TGB3	TGB1—has nucleic acid-binding activity at its N-terminal TGB1—contains ATP/GTP-dependent SF1 helicase type consensus sequence motifs TGB1, TGB2 and TGB3 are localized on ER-derived peripheral membrane bodies	PD-gating (increase SEL) TGB 2 and 3 facilitate the targeting of TGB1 to PD-associated peripheral punctate bodies	NK	NK	[12]
*Tombusviridae/ Umbravirus*	PEMV-2	P26 and P27	Bind RNA, interact with PEMV-1 *Enamovirus* during natural coinfections and assist its movement Protect viral and host transcript from nonsense-mediated decay	Tubule formation	NK	NK	[95]
*Caulimoviridae /Caulimovirus*	CaMV	38 kDa MP (P1)	Binds RNA	Tubule formation	P6—forms virus inclusion bodies that serve as translation sites for other proteins, including MP P3—for MP-CP interaction at PD	PRA1—vesicle trafficking regulation AtSRC2.2 and PDLPs—recruitment of MP to PD CHUP1—virion delivery to PD MP17—a rab acceptor-like vesicle-associated protein and PME-putative role in intercellular movement. PDLPs—recruitment of MP to PD	[96,97,98,99]
*Secoviridae/Nepovirus*	GFLV	38 kDa MP	Binds RNA	Tubule formation	CP	KNOLLE—vesicle trafficking of MP Myosin—MP delivery to PD Calreticulin and PDLPs—recruitment of MP to PD	[100,101,102]
*Comoviridae/* *Comovirus*	CPMV	48 kDa MP	Has large CP, GTP, ssRNA, ssDNA-binding regions	Tubule formation	Large CP (CPL)-37 kDa	PDLPs-recruitment of MP to PD	[103,104,105,106,107]
*Bunyaviridae/* *Tospovirus*	TSWV	NSm protein	Binds ssRNA Avirulence determinant for Sw-5 and RTSW resistance gene Associated with the ER membrane	Tubule formation	TSWV N—protein-recognition of nucleocapsid structures	DNA J-like chaperone proteins, e.g., AtA39—may act as a putative molecular motor for intercellular transport NbSGT1—a molecular co-chaperone, interacts with NSm for intercellular and systemic infection	[96,97,98,99,108,109,110]
*Tombusviridae/* *Umbravirus*	GRV	28 kDa, MP (P4)	PD-localized Binds to both ssDNA and ssRNA	Tubule formation	ORF3 protein assist for vRNP formation for cell-to-cell movement	Fibrillarin—vRNP complex formation for transport, interacts with virus ORF3 protein	[111,112]
*Bromoviridae/Alfamovirus*	AMV	32 kDa MP (P3)	Bind RNA PD-localized ER-associated	PD-gating (increases SEL) Tubule formation	CP	Patellin 3 (atPATL3) and Patellin 6 (atPATL6) —inhibitory effect on intercellular movement Host kinases—for MP phosphorylation	[113]
*Bromoviridae/* *Bromovirus*	BMV	32 kDa MP (3a)	Binds RNA PD-localized	PD-gating (increases SEL)/ Tubule formation (BMV-MI?)	CP (BMV-MI strain)	NbNACa1—PD localization	[114,115,116]
*Bromoviridae/* *Cucumovirus*	CMV	32 to 36 kDa MP (3a) 2b-for long-distance movement	Binds RNA PD located suppresses the PAMP-triggered immune responses of the host 2b is a VSR	PD-gating (increases SEL)/ Tubule formation	CP (for tubule formation?)	Ascorbate oxidase—movement of vRNP-MP to PD Actin—PD-gating RIO kinase—MP phosphorylation	[117,118,119,120]
*Bromoviridae/Ilarvirus*	PNRSV	32 to 36 kDa MP (3a)	Binds RNA PD located	PD-gating (increased SEL)/ Tubule formation	CP	NK	[121]

^a^ The TGB proteins of GRSPaV (*Foveavirus*) is highly similar to their counterparts in potato virus X (PVX) [94]. Abbreviations: ADV—alfalfa dwarf virus; AMV—alfalfa mosaic virus; AtSRC2.2—*Arabidopsis thaliana* soybean response to cold; BCNMV—bean common mosaic necrosis virus; BMV—brome mosaic virus; BNYVV—beet necrotic yellow vein virus; BSMV—barley stripe mosaic virus; BYDV—barley yellow dwarf virus; BYV—beet yellows virus; CarMV—carnation mottle virus; CHUP1—chloroplast unusual positioning 1; CK2—casein kinase 2; CMV—cucumber mosaic virus; Eb1a—end-binding protein 1a; EMDV—eggplant mottled dwarf virus; GRSPaV—Grapevine rupestris stem pitting-associated virus; HGSV—hibiscus green spot virus; HSP70h—heat shock protein 70 homolog; LNYV—lettuce necrotic yellows virus; LMV—lettuce mosaic virus; LIYV—lettuce infectious yellows virus; MBF-1—multiprotein bridging factor 1; MPB2C—movement protein-binding 2C; MNSV—melon necrotic spot virus; MMV—maize mosaic virus MFSV—maize fine streak virus; NK—not known; NtMPIP1—*Nicotiana tabacum* MP interacting protein 1; PAMP—pathogen-associated molecular patterns; PAPK—plasmodesmal-associated protein kinase; PCD—programmed cell death; PEMV-2—pea entaion mosaic virus-2; PeVYV—pepper vein yellows virus; PFBV—pelargonium flower break *Carmovirus*; PLRV—potato leafroll virus; PME—pectin methylesterase; PNRSV—prunus necrotic ringspot virus; PRA-1—prenylated rab acceptor1; PVX—potato virus X; PYDV—potato yellow dwarf virus; RCNMV—red clover necrotic mosaic virus; RIO kinase—Serine/threonine–protein kinase; RTSW—TSWV resistance locus; RYSV—rice yellow stunt virus; SCD-2—stomatal cytokinesis defective 2; SMV—soybean mosaic virus; SYNV—Sonchus yellow net virus; TBSV—tomato bushy stunt virus; TCV—turnip crinkle virus; TEV—tobacco etch virus; TMV—tobacco mosaic virus; ToLCNDV—tomato leaf curl New Delhi virus; TuYV—turnip yellows virus; TYMaV—tomato yellow mottle-associated virus; vRNA—viral RNA; vRNP—viral ribonucleoprotein; VSR—viral suppressor of RNA silencing.

## Data Availability

Not applicable.

## References

[1] Navarro J.A., Sanchez-Navarro J.A., Pallas V. (2019). Key checkpoints in the movement of plant viruses through the host. Advances in Virus Research.

[2] Kappagantu M., Collum T.D., Dardick C., Culver J.N. (2020). Viral Hacks of the Plant Vasculature: The Role of Phloem Alterations in Systemic Virus Infection. Annu. Rev. Virol..

[3] Terry B.R., Robards A.W. (1987). Hydrodynamic radius alone governs the mobility of molecules through plasmodesmata. Planta.

[4] Liarzi O., Epel B.L. (2005). Development of a quantitative tool for measuring changes in the coefficient of conductivity of plasmodesmata induced by developmental, biotic, and abiotic signals. Protoplasma.

[5] Lucas W.J. (2006). Plant viral movement proteins: Agents for cell-to-cell trafficking of viral genomes. Virology.

[6] Hong J.-S., Ju H.-J. (2017). The plant cellular systems for plant virus movement. Plant Pathol. J..

[7] Schoelz J.E., Angel C.A., Nelson R.S., Leisner S.M. (2016). A model for intracellular movement of Cauliflower mosaic virus: The concept of the mobile virion factory. J. Exp. Bot..

[8] Kumar D., Kumar R., Hyun T.K., Kim J.-Y. (2015). Cell-to-cell movement of viruses via plasmodesmata. J. Plant Res..

[9] Heinlein M. (2015). Plant virus replication and movement. Virology.

[10] Pitzalis N., Heinlein M. (2018). The roles of membranes and associated cytoskeleton in plant virus replication and cell-to-cell movement. J. Exp. Bot..

[11] Reagan B.C., Burch-Smith T.M. (2020). Viruses reveal the secrets of plasmodesmal cell biology. Mol. Plant-Microbe Interact..

[12] Morozov S.Y., Solovyev A.G. (2020). Small hydrophobic viral proteins involved in intercellular movement of diverse plant virus genomes. Aims Microbiol..

[13] Levy A., Tilsner J. (2020). Creating contacts between replication and movement at plasmodesmata–a role for membrane contact sites in plant virus infections?. Front. Plant Sci..

[14] Wu X., Cheng X. (2020). Intercellular movement of plant RNA viruses: Targeting replication complexes to the plasmodesma for both accuracy and efficiency. Traffic.

[15] Deom C.M., Lapidot M., Beachy R.N. (1992). Plant virus movement proteins. Cell.

[16] Kellmann J.-W. (2001). Identification of plant virus movement-host protein interactions. Zeitschrift Für Naturforschung C.

[17] Folimonova S.Y., Tilsner J. (2018). Hitchhikers, highway tolls and roadworks: The interactions of plant viruses with the phloem. Curr. Opin. Plant Biol..

[18] Verchot-Lubicz J., Torrance L., Solovyev A.G., Morozov S.Y., Jackson A.O., Gilmer D. (2010). Varied movement strategies employed by triple gene block–encoding viruses. Mol. Plant-Microbe Interact..

[19] Nicolas W.J., Grison M.S., Bayer E.M. (2018). Shaping intercellular channels of plasmodesmata: The structure-to-function missing link. J. Exp. Bot..

[20] Dorokhov Y.L., Ershova N.M., Sheshukova E.V., Komarova T.V. (2019). Plasmodesmata Conductivity Regulation: A Mechanistic Model. Plants.

[21] Radford J.E., White R.G. (1998). Localization of a myosin-like protein to plasmodesmata. Plant J..

[22] Zavaliev R., Levy A., Gera A., Epel B.L. (2013). Subcellular dynamics and role of Arabidopsis β-1, 3-glucanases in cell-to-cell movement of tobamoviruses. Mol. Plant-Microbe Interact..

[23] Chen M.-H., Tian G.-W., Gafni Y., Citovsky V. (2005). Effects of calreticulin on viral cell-to-cell movement. Plant Physiol..

[24] Baluška F., Šamaj J., Napier R., Volkmann D. (1999). Maize calreticulin localizes preferentially to plasmodesmata in root apex. Plant J..

[25] Diao M., Ren S., Wang Q., Qian L., Shen J., Liu Y., Huang S. (2018). Arabidopsis formin 2 regulates cell-to-cell trafficking by capping and stabilizing actin filaments at plasmodesmata. Elife.

[26] Iswanto A.B.B., Kim J.-Y. (2017). Lipid raft, regulator of plasmodesmal callose homeostasis. Plants.

[27] Lee J.-Y., Yoo B.-C., Rojas M.R., Gomez-Ospina N., Staehelin L.A., Lucas W.J. (2003). Selective trafficking of non-cell-autonomous proteins mediated by NtNCAPP1. Science.

[28] Simpson C., Thomas C., Findlay K., Bayer E., Maule A.J. (2009). An Arabidopsis GPI-anchor plasmodesmal neck protein with callose binding activity and potential to regulate cell-to-cell trafficking. Plant Cell.

[29] Lim G.-H., Shine M.B., de Lorenzo L., Yu K., Cui W., Navarre D., Hunt A.G., Lee J.-Y., Kachroo A., Kachroo P. (2016). Plasmodesmata localizing proteins regulate transport and signaling during systemic acquired immunity in plants. Cell Host Microbe.

[30] Raffaele S., Bayer E., Lafarge D., Cluzet S., Retana S.G., Boubekeur T., Leborgne-Castel N., Carde J.-P., Lherminier J., Noirot E. (2009). Remorin, a solanaceae protein resident in membrane rafts and plasmodesmata, impairs potato virus X movement. Plant Cell.

[31] Burch-Smith T.M., Cui Y., Zambryski P.C. (2012). Reduced levels of class 1 reversibly glycosylated polypeptide increase intercellular transport via plasmodesmata. Plant Signal. Behav..

[32] Yuan C., Lazarowitz S.G., Citovsky V. (2018). The plasmodesmal localization signal of TMV MP is recognized by plant synaptotagmin SYTA. MBio.

[33] Diao M., Huang S. (2021). An Update on the Role of the Actin Cytoskeleton in Plasmodesmata: A Focus on Formins. Front. Plant Sci..

[34] Nishiguchi M., Motoyoshi F., Oshima N. (1978). Behaviour of a temperature sensitive strain of tobacco mosaic virus in tomato leaves and protoplasts. J. Gen. Virol..

[35] Wolf S., Lucas W.J., Deom C.M., Beachy R.N. (1989). Movement protein of tobacco mosaic virus modifies plasmodesmatal size exclusion limit. Science.

[36] Ueki S., Spektor R., Natale D.M., Citovsky V. (2010). ANK, a host cytoplasmic receptor for the Tobacco mosaic virus cell-to-cell movement protein, facilitates intercellular transport through plasmodesmata. PLoS Pathog..

[37] Melcher U. (2000). The ‘30K’superfamily of viral movement proteins. J. Gen. Virol..

[38] Moore P.J., Fenczik C.A., Deom C.M., Beachy R.N. (1992). Developmental changes in plasmodesmata in transgenic tobacco expressing the movement protein of tobacco mosaic virus. Protoplasma.

[39] Beachy R.N., Heinlein M. (2000). Role of P30 in replication and spread of TMV. Traffic.

[40] Peiró A., Martínez-Gil L., Tamborero S., Pallás V., Sánchez-Navarro J.A., Mingarro I. (2014). The Tobacco mosaic virus movement protein associates with but does not integrate into biological membranes. J. Virol..

[41] Guenoune-Gelbart D., Elbaum M., Sagi G., Levy A., Epel B.L. (2008). Tobacco mosaic virus (TMV) replicase and movement protein function synergistically in facilitating TMV spread by lateral diffusion in the plasmodesmal desmotubule of Nicotiana benthamiana. Mol. Plant-Microbe Interact..

[42] Kawakami S., Watanabe Y., Beachy R.N. (2004). Tobacco mosaic virus infection spreads cell to cell as intact replication complexes. Proc. Natl. Acad. Sci. USA.

[43] Matsushita Y., Hanazawa K., Yoshioka K., Oguchi T., Kawakami S., Watanabe Y., Nishiguchi M., Nyunoya H. (2000). In vitro phosphorylation of the movement protein of tomato mosaic tobamovirus by a cellular kinase. J. Gen. Virol..

[44] Matsushita Y., Ohshima M., Yoshioka K., Nishiguchi M., Nyunoya H. (2003). The catalytic subunit of protein kinase CK2 phosphorylates in vitro the movement protein of Tomato mosaic virus. J. Gen. Virol..

[45] Lee J.-Y., Taoka K., Yoo B.-C., Ben-Nissan G., Kim D.-J., Lucas W.J. (2005). Plasmodesmal-associated protein kinase in tobacco and Arabidopsis recognizes a subset of non-cell-autonomous proteins. Plant Cell.

[46] Trutnyeva K., Bachmaier R., Waigmann E. (2005). Mimicking carboxyterminal phosphorylation differentially effects subcellular distribution and cell-to-cell movement of Tobacco mosaic virus movement protein. Virology.

[47] Yuan C., Lazarowitz S.G., Citovsky V. (2016). Identification of a functional plasmodesmal localization signal in a plant viral cell-to-cell-movement protein. MBio.

[48] Liu Y., Huang C., Zeng J., Yu H., Li Y., Yuan C. (2020). Identification of two additional plasmodesmata localization domains in the tobacco mosaic virus cell-to-cell-movement protein. Biochem. Biophys. Res. Commun..

[49] Levy A., Zheng J.Y., Lazarowitz S.G. (2015). Synaptotagmin SYTA forms ER-plasma membrane junctions that are recruited to plasmodesmata for plant virus movement. Curr. Biol..

[50] Ishikawa K., Tamura K., Fukao Y., Shimada T. (2020). Structural and functional relationships between plasmodesmata and plant endoplasmic reticulum–plasma membrane contact sites consisting of three synaptotagmins. New Phytol..

[51] Harries P.A., Park J.-W., Sasaki N., Ballard K.D., Maule A.J., Nelson R.S. (2009). Differing requirements for actin and myosin by plant viruses for sustained intercellular movement. Proc. Natl. Acad. Sci. USA.

[52] Amsbury S., Kirk P., Benitez-Alfonso Y. (2018). Emerging models on the regulation of intercellular transport by plasmodesmata-associated callose. J. Exp. Bot..

[53] Chen M., Sheng J., Hind G., Handa A.K., Citovsky V. (2000). Interaction between the tobacco mosaic virus movement protein and host cell pectin methylesterases is required for viral cell-to-cell movement. Embo J..

[54] Chen M., Citovsky V. (2003). Systemic movement of a tobamovirus requires host cell pectin methylesterase. Plant J..

[55] Oparka K.J., Prior D.A.M., Cruz S.S., Padgett H.S., Beachy R.N. (1997). Gating of epidermal plasmodesmata is restricted to the leading edge of expanding infection sites of tobacco mosaic virus (TMV). Plant J..

[56] Komarova T.V., Skulachev M.V., Ivanov P.A., Klyushin A.G., Dorokhov Y.L., Atabekov J.G. (2003). Internal ribosome entry site from crucifer tobamovirus promotes initiation of translation in Escherichia coli. Dokl. Biochem. Biophys..

[57] Zvereva S.D., Ivanov P.A., Skulachev M.V., Klyushin A.G., Dorokhov Y.L., Atabekov J.G. (2004). Evidence for contribution of an internal ribosome entry site to intercellular transport of a tobamovirus. J. Gen. Virol..

[58] Dorokhov Y.L., Ivanov P.A., Komarova T.V., Skulachev M.V., Atabekov J.G. (2006). An internal ribosome entry site located upstream of the crucifer-infecting tobamovirus coat protein (CP) gene can be used for CP synthesis in vivo. J. Gen. Virol..

[59] Vogler H., Kwon M.-O., Dang V., Sambade A., Fasler M., Ashby J., Heinlein M. (2008). Tobacco mosaic virus movement protein enhances the spread of RNA silencing. PLoS Pathog..

[60] Xiong Z., Kim K.H., Giesman-Cookmeyer D., Lommel S.A. (1993). The roles of the red clover necrotic mosaic virus capsid and cell-to-cell movement proteins in systemic infection. Virology.

[61] Tremblay D., Vaewhongs A.A., Turner K.A., Sit T.L., Lommel S.A. (2005). Cell wall localization of Red clover necrotic mosaic virus movement protein is required for cell-to-cell movement. Virology.

[62] Powers J.G., Sit T.L., Heinsohn C., George C.G., Kim K.-H., Lommel S.A. (2008). The Red clover necrotic mosaic virus RNA-2 encoded movement protein is a second suppressor of RNA silencing. Virology.

[63] Kaido M., Tsuno Y., Mise K., Okuno T. (2009). Endoplasmic reticulum targeting of the Red clover necrotic mosaic virus movement protein is associated with the replication of viral RNA1 but not that of RNA2. Virology.

[64] Kaido M., Funatsu N., Tsuno Y., Mise K., Okuno T. (2011). Viral cell-to-cell movement requires formation of cortical punctate structures containing Red clover necrotic mosaic virus movement protein. Virology.

[65] Cao M., Ye X., Willie K., Lin J., Zhang X., Redinbaugh M.G., Simon A.E., Morris T.J., Qu F. (2010). The capsid protein of Turnip crinkle virus overcomes two separate defense barriers to facilitate systemic movement of the virus in Arabidopsis. J. Virol..

[66] Martínez-Gil L., Johnson A.E., Mingarro I. (2010). Membrane insertion and biogenesis of the Turnip crinkle virus p9 movement protein. J. Virol..

[67] Vilar M., Saurí A., Marcos J.F., Mingarro I., Pérez-Payá E. (2005). Transient structural ordering of the RNA-binding domain of carnation mottle virus p7 movement protein modulates nucleic acid binding. Chembiochem.

[68] Martínez-Turiño S., Hernández C. (2011). A membrane-associated movement protein of Pelargonium flower break virus shows RNA-binding activity and contains a biologically relevant leucine zipper-like motif. Virology.

[69] Genovés A., Navarro J.A., Pallás V. (2010). The intra-and intercellular movement of Melon necrotic spot virus (MNSV) depends on an active secretory pathway. Mol. Plant-Microbe Interact..

[70] Serra-Soriano M., Antonio Navarro J., Pallás V. (2017). Dissecting the multifunctional role of the N-terminal domain of the Melon necrotic spot virus coat protein in RNA packaging, viral movement and interference with antiviral plant defence. Mol. Plant Pathol..

[71] Lazareva E.A., Lezzhov A.A., Komarova T.V., Morozov S.Y., Heinlein M., Solovyev A.G. (2017). A novel block of plant virus movement genes. Mol. Plant Pathol..

[72] Lazareva E.A., Lezzhov A.A., Chergintsev D.A., Golyshev S.A., Dolja V.V., Morozov S.Y., Heinlein M., Solovyev A.G. (2021). Reticulon-like properties of a plant virus-encoded movement protein. New Phytol..

[73] Chu M., Park J.-W., Scholthof H.B. (1999). Separate regions on the tomato bushy stunt virus p22 protein mediate cell-to-cell movement versus elicitation of effective resistance responses. Mol. Plant-Microbe Interact..

[74] Agranovsky A.A. (2016). Closteroviruses: Molecular biology, evolution and interactions with cells. Plant Viruses: Evolution and Management.

[75] Qiao W., Medina V., Kuo Y.-W., Falk B.W. (2018). A distinct, non-virion plant virus movement protein encoded by a crinivirus essential for systemic infection. MBio.

[76] Cui X., Yaghmaiean H., Wu G., Wu X., Chen X., Thorn G., Wang A. (2017). The C-terminal region of the Turnip mosaic virus P3 protein is essential for viral infection via targeting P3 to the viral replication complex. Virology.

[77] Dai Z., He R., Bernards M.A., Wang A. (2020). The cis-expression of the coat protein of turnip mosaic virus is essential for viral intercellular movement in plants. Mol. Plant Pathol..

[78] Rojas M.R., Zerbini F.M., Allison R.F., Gilbertson R.L., Lucas W.J. (1997). Capsid protein and helper component-proteinase function as potyvirus cell-to-cell movement proteins. Virology.

[79] Cheng G., Yang Z., Zhang H., Zhang J., Xu J. (2020). Remorin interacting with PCaP1 impairs Turnip mosaic virus intercellular movement but is antagonised by VPg. New Phytol..

[80] Chai M., Wu X., Liu J., Fang Y., Luan Y., Cui X., Zhou X., Wang A., Cheng X. (2020). P3N-PIPO interacts with P3 via the shared N-terminal domain to recruit viral replication vesicles for cell-to-cell movement. J. Virol..

[81] Lee L., Palukaitis P., Gray S.M. (2002). Host-dependent requirement for the Potato leafroll virus 17-kda protein in virus movement. Mol. Plant-Microbe Interact..

[82] DeBlasio S.L., Xu Y., Johnson R.S., Rebelo A.R., MacCoss M.J., Gray S.M., Heck M. (2018). The interaction dynamics of two potato leafroll virus movement proteins affects their localization to the outer membranes of mitochondria and plastids. Viruses.

[83] Li S., Su X., Luo X., Zhang Y., Zhang D., Du J., Zhang Z., OuYang X., Zhang S., Liu Y. (2020). First evidence showing that Pepper vein yellows virus P4 protein is a movement protein. BMC Microbiol..

[84] Fusaro A.F., Barton D.A., Nakasugi K., Jackson C., Kalischuk M.L., Kawchuk L.M., Vaslin M.F.S., Correa R.L., Waterhouse P.M. (2017). The luteovirus P4 movement protein is a suppressor of systemic RNA silencing. Viruses.

[85] Kleinow T., Happle A., Kober S., Linzmeier L., Rehm T.M., Fritze J., Buchholz P.C.F., Kepp G., Jeske H., Wege C. (2020). Phosphorylations of the Abutilon Mosaic Virus Movement Protein Affect Its Self-Interaction, Symptom Development, Viral DNA Accumulation, and Host Range. Front. Plant Sci..

[86] Lee C., Zheng Y., Chan C., Ku H., Chang C., Jan F. (2020). A single amino acid substitution in the movement protein enables the mechanical transmission of a geminivirus. Mol. Plant Pathol..

[87] Mann K.S., Bejerman N., Johnson K.N., Dietzgen R.G. (2016). Cytorhabdovirus P3 genes encode 30K-like cell-to-cell movement proteins. Virology.

[88] Zhou X., Lin W., Sun K., Wang S., Zhou X., Jackson A.O., Li Z. (2019). Specificity of plant rhabdovirus cell-to-cell movement. J. Virol..

[89] Solovyev A., Kalinina N., Morozov S. (2012). Recent advances in research of plant virus movement mediated by triple gene block. Front. Plant Sci..

[90] Lee M.Y., Yan L., Gorter F.A., Kim B.Y.T., Cui Y., Hu Y., Yuan C., Grindheim J., Ganesan U., Liu Z. (2012). Brachypodium distachyon line Bd3-1 resistance is elicited by the barley stripe mosaic virus triple gene block 1 movement protein. J. Gen. Virol..

[91] Perraki A., Binaghi M., Mecchia M.A., Gronnier J., German-Retana S., Mongrand S., Bayer E., Zelada A.M., Germain V. (2014). StRemorin1. 3 hampers Potato virus X TGBp1 ability to increase plasmodesmata permeability, but does not interfere with its silencing suppressor activity. FEBS Lett..

[92] Mathioudakis M.M., Veiga R.S.L., Canto T., Medina V., Mossialos D., Makris A.M., Livieratos I. (2013). P epino mosaic virus triple gene block protein 1 (TGBp1) interacts with and increases tomato catalase 1 activity to enhance virus accumulation. Mol. Plant Pathol..

[93] Wu X., Liu J., Chai M., Wang J., Li D., Wang A., Cheng X. (2019). The Potato virus X TGBp2 protein plays dual functional roles in viral replication and movement. J. Virol..

[94] Mann K., Meng B. (2013). The triple gene block movement proteins of a grape virus in the genus Foveavirus confer limited cell-to-cell spread of a mutant Potato virus X. Virus Genes.

[95] May J.P., Johnson P.Z., Ilyas M., Gao F., Simon A.E. (2020). The Multifunctional Long-Distance Movement Protein of Pea Enation Mosaic Virus 2 Protects Viral and Host Transcripts from Nonsense-Mediated Decay. Mbio.

[96] Kasteel D.T.J., Perbal M.-C., Boyer J.-C., Wellink J., Goldbach R.W., Maule A.J., Van Lent J.W.M. (1996). The movement proteins of cowpea mosaic virus and cauliflower mosaic virus induce tubular structures in plant and insect cells. J. Gen. Virol..

[97] Thomas C.L., Perbal C., Maule A.J. (1993). A mutation of cauliflower mosaic virus gene I interferes with virus movement but not virus replication. Virology.

[98] Amari K., Boutant E., Hofmann C., Schmitt-Keichinger C., Fernandez-Calvino L., Didier P., Lerich A., Mutterer J., Thomas C.L., Heinlein M. (2010). A family of plasmodesmal proteins with receptor-like properties for plant viral movement proteins. PLoS Pathog..

[99] Kitajima E.W., Lauritis J.A. (1969). Plant virions in plasmodesmata. Virology.

[100] Ritzenthaler C., Schmit A.C., Michler P., Stussi-Garaud C., Pinck L. (1995). Grapevine fanleaf nepovirus P38 putative movement protein is located on tubules in vivo. Mol. Plant-Microbe Interact..

[101] Laporte C., Vetter G., Loudes A.-M., Robinson D.G., Hillmer S., Stussi-Garaud C., Ritzenthaler C. (2003). Involvement of the secretory pathway and the cytoskeleton in intracellular targeting and tubule assembly of Grapevine fanleaf virus movement protein in tobacco BY-2 cells. Plant Cell.

[102] Belin C., Schmitt C., Gaire F., Walter B., Demangeat G., Pinck L. (1999). The nine C-terminal residues of the grapevine fanleaf nepovirus movement protein are critical for systemic virus spread. J. Gen. Virol..

[103] Lekkerkerker A., Wellink J., Yuan P., Van Lent J., Goldbach R., Van Kammen A.B. (1996). Distinct functional domains in the cowpea mosaic virus movement protein. J. Virol..

[104] Bertens P., Wellink J., Goldbach R., van Kammen A. (2000). Mutational analysis of the cowpea mosaic virus movement protein. Virology.

[105] Bertens P., Heijne W., Van der Wel N., Wellink J., Van Kammen A. (2003). Studies on the C-terminus of the Cowpea mosaic virus movement protein. Arch. Virol..

[106] Pouwels J., Kornet N., van Bers N., Guighelaar T., van Lent J., Bisseling T., Wellink J. (2003). Identification of distinct steps during tubule formation by the movement protein of Cowpea mosaic virus. J. Gen. Virol..

[107] Pouwels J., van der Velden T., Willemse J., Borst J.W., van Lent J., Bisseling T., Wellink J. (2004). Studies on the origin and structure of tubules made by the movement protein of Cowpea mosaic virus. J. Gen. Virol..

[108] Storms M.M.H., Kromelink R., Peters D., Van Lent J.W.M., Goldbach R.O.B.W. (1995). The nonstructural NSm protein of tomato spotted wilt virus induces tubular structures in plant and insect cells. Virology.

[109] Liu C., Ye L., Lang G., Zhang C., Hong J., Zhou X. (2011). The VP37 protein of Broad bean wilt virus 2 induces tubule-like structures in both plant and insect cells. Virus Res..

[110] Wijkamp I., van Lent J., Kormelink R., Goldbach R., Peters D. (1993). Multiplication of tomato spotted wilt virus in its insect vector, Frankliniella occidentalis. J. Gen. Virol..

[111] Ryabov E.V., Oparka K.J., Santa Cruz S., Robinson D.J., Taliansky M.E. (1998). Intracellular location of two groundnut rosette umbravirus proteins delivered by PVX and TMV vectors. Virology.

[112] Nurkiyanova K.M., Ryabov E.V., Kalinina N.O., Fan Y., Andreev I., Fitzgerald A.G., Palukaitis P., Taliansky M. (2001). Umbravirus-encoded movement protein induces tubule formation on the surface of protoplasts and binds RNA incompletely and non-cooperatively. J. Gen. Virol..

[113] Tenllado F., Bol J.F. (2000). Genetic dissection of the multiple functions of alfalfa mosaic virus coat protein in viral RNA replication, encapsidation, and movement. Virology.

[114] Kasteel D.T., Van der Wel N.N., Jansen K.A., Goldbach R.W., Van Lent J.W. (1997). Tubule-forming capacity of the movement proteins of alfalfa mosaic virus and brome mosaic virus. J. Gen. Virol..

[115] Takeda A., Kaido M., Okuno T., Mise K. (2004). The C terminus of the movement protein of Brome mosaic virus controls the requirement for coat protein in cell-to-cell movement and plays a role in long-distance movement. J. Gen. Virol..

[116] Kaido M., Inoue Y., Takeda Y., Sugiyama K., Takeda A., Mori M., Tamai A., Meshi T., Okuno T., Mise K. (2007). Downregulation of the NbNACa1 gene encoding a movement-protein-interacting protein reduces cell-to-cell movement of Brome mosaic virus in Nicotiana benthamiana. Mol. Plant-Microbe Interact..

[117] Canto T., Palukaitis P. (1999). Are tubules generated by the 3a protein necessary for cucumber mosaic virus movement?. Mol. Plant-Microbe Interact..

[118] Palukaitis P., García-Arenal F. (2003). Cucumoviruses. Adv. Virus Res..

[119] Nagano H., Mise K., Furusawa I., Okuno T. (2001). Conversion in the requirement of coat protein in cell-to-cell movement mediated by the cucumber mosaic virus movement protein. J. Virol..

[120] Andreev I.A., Kim S.H., Kalinina N.O., Rakitina D.V., Fitzgerald A.G., Palukaitis P., Taliansky M.E. (2004). Molecular interactions between a plant virus movement protein and RNA: Force spectroscopy investigation. J. Mol. Biol..

[121] Herranz M.C., Pallas V. (2004). RNA-binding properties and mapping of the RNA-binding domain from the movement protein of Prunus necrotic ringspot virus. J. Gen. Virol..

[122] Waigmann E., Lucas W.J., Citovsky V., Zambryski P. (1994). Direct functional assay for tobacco mosaic virus cell-to-cell movement protein and identification of a domain involved in increasing plasmodesmal permeability. Proc. Natl. Acad. Sci. USA.

[123] Epel B.L. (2009). Plant viruses spread by diffusion on ER-associated movement-protein-rafts through plasmodesmata gated by viral induced host beta-1,3-glucanases. Semin Cell Dev. Biol..

[124] Zavaliev R., Sagi G., Gera A., Epel B.L. (2010). The constitutive expression of Arabidopsis plasmodesmal-associated class 1 reversibly glycosylated polypeptide impairs plant development and virus spread. J. Exp. Bot..

[125] Adkar-Purushothama C.R., Brosseau C., Giguère T., Sano T., Moffett P., Perreault J.-P. (2015). Small RNA derived from the virulence modulating region of the potato spindle tuber viroid silences callose synthase genes of tomato plants. Plant Cell.

[126] Cui W., Lee J.-Y. (2016). Arabidopsis callose synthases CalS1/8 regulate plasmodesmal permeability during stress. Nat. Plants.

[127] Yan D., Yadav S.R., Paterlini A., Nicolas W.J., Petit J.D., Brocard L., Belevich I., Grison M.S., Vaten A., Karami L. (2019). Sphingolipid biosynthesis modulates plasmodesmal ultrastructure and phloem unloading. Nat. Plants.

[128] Adams M.J., Adkins S., Bragard C., Gilmer D., Li D., MacFarlane S.A., Wong S.-M., Melcher U., Ratti C., Ryu K.H. (2017). ICTV virus taxonomy profile: Virgaviridae. J. Gen. Virol..

[129] Rojas M.R., Maliano M.R., de Souza J.O., Vasquez-Mayorga M., de Macedo M.A., Ham B.-K., Gilbertson R.L. (2016). Cell-to-cell movement of plant viruses: A diversity of mechanisms and strategies. Current Research Topics in Plant Virology.

[130] Sheshukova E.V., Ershova N.M., Kamarova K.A., Dorokhov Y.L., Komarova T.V. (2020). The Tobamoviral Movement Protein: A “Conditioner” to Create a Favorable Environment for Intercellular Spread of Infection. Front. Plant Sci..

[131] Curin M., Ojangu E.-L., Trutnyeva K., Ilau B., Truve E., Waigmann E. (2007). MPB2C, a microtubule-associated plant factor, is required for microtubular accumulation of tobacco mosaic virus movement protein in plants. Plant Physiol..

[132] Kleinow T., Tanwir F., Kocher C., Krenz B., Wege C., Jeske H. (2009). Expression dynamics and ultrastructural localization of epitope-tagged Abutilon mosaic virus nuclear shuttle and movement proteins in Nicotiana benthamiana cells. Virology.

[133] Levy A., Tzfira T. (2010). Bean dwarf mosaic virus: A model system for the study of viral movement. Mol. Plant Pathol..

[134] Kumar R.V. (2019). Plant antiviral immunity against geminiviruses and viral counter-defense for survival. Front. Microbiol..

[135] Lazarowitz S.G., Beachy R.N. (1999). Viral movement proteins as probes for intracellular and intercellular trafficking in plants. Plant Cell.

[136] van Lent J., Storms M., van der Meer F., Wellink J., Goldbach R. (1991). Tubular structures involved in movement of cowpea mosaic virus are also formed in infected cowpea protoplasts. J. Gen. Virol..

[137] Carluccio A.V., Zicca S., Stavolone L. (2014). Hitching a ride on vesicles: Cauliflower mosaic virus movement protein trafficking in the endomembrane system. Plant Physiol..

[138] Taliansky M.E., Robinson D.J. (2003). Molecular biology of umbraviruses: Phantom warriors. J. Gen. Virol..

[139] Ritzenthaler C., Hofmann C., Waigmann E., Heinlein M. (2007). Tubule-Guided Movement of Plant Viruses. Viral Transport in Plants.

[140] Carvalho C.M., Wellink J., Ribeiro S.G., Goldbach R.W., Van Lent J.W.M. (2003). The C-terminal region of the movement protein of Cowpea mosaic virus is involved in binding to the large but not to the small coat protein. J. Gen. Virol..

[141] Liu C., Meng C., Xie L., Hong J., Zhou X. (2009). Cell-to-cell trafficking, subcellular distribution, and binding to coat protein of Broad bean wilt virus 2 VP37 protein. Virus Res..

[142] Zheng H., Wang G., Zhang L. (1997). Alfalfa mosaic virus movement protein induces tubules in plant protoplasts. Mol. Plant-Microbe Interact..

[143] Callaway A., Giesman-Cookmeyer D., Gillock E.T., Sit T.L., Lommel S.A. (2001). The multifunctional capsid proteins of plant RNA viruses. Annu. Rev. Phytopathol..

[144] Rao A.L.N., Cooper B. (2006). Capsid protein gene and the type of host plant differentially modulate cell-to-cell movement of cowpea chlorotic mottle virus. Virus Genes.

[145] Sasaki N., Arimoto M., Nagano H., Mori M., Kaido M., Mise K., Okuno T. (2003). The movement protein gene is involved in the virus-specific requirement of the coat protein in cell-to-cell movement of bromoviruses. Arch. Virol..

[146] Muhammad T., Zhang F., Zhang Y., Liang Y. (2019). RNA interference: A natural immune system of plants to counteract biotic stressors. Cells.

[147] Pumplin N., Voinnet O. (2013). RNA silencing suppression by plant pathogens: Defence, counter-defence and counter-counter-defence. Nat. Rev. Microbiol..

[148] Schwartz M., Chen J., Janda M., Sullivan M., den Boon J., Ahlquist P. (2002). A positive-strand RNA virus replication complex parallels form and function of retrovirus capsids. Mol. Cell.

[149] Díaz-Pendón J.A., Ding S.W. (2008). Direct and indirect roles of viral suppressors of RNA silencing in pathogenesis. Annu. Rev. Phytopathol.

[150] Chiu M., Chen I., Baulcombe D.C., Tsai C. (2010). The silencing suppressor P25 of Potato virus X interacts with Argonaute1 and mediates its degradation through the proteasome pathway. Mol. Plant Pathol..

[151] Wu J., Du Z., Wang C., Cai L., Hu M., Lin Q., Wu Z., Li Y., Xie L. (2010). Identification of Pns6, a putative movement protein of RRSV, as a silencing suppressor. Virol. J..

[152] Yaegashi H., Takahashi T., Isogai M., Kobori T., Ohki S., Yoshikawa N. (2007). Apple chlorotic leaf spot virus 50 kDa movement protein acts as a suppressor of systemic silencing without interfering with local silencing in Nicotiana benthamiana. J. Gen. Virol..

[153] Renovell A., Vives M.C., Ruiz-Ruiz S., Navarro L., Moreno P., Guerri J. (2012). The Citrus leaf blotch virus movement protein acts as silencing suppressor. Virus Genes.

[154] Zvereva A.S., Golyaev V., Turco S., Gubaeva E.G., Rajeswaran R., Schepetilnikov M.V., Srour O., Ryabova L.A., Boller T., Pooggin M.M. (2016). Viral protein suppresses oxidative burst and salicylic acid-dependent autophagy and facilitates bacterial growth on virus-infected plants. New Phytol..

[155] Csorba T., Kontra L., Burgyán J. (2015). Viral silencing suppressors: Tools forged to fine-tune host-pathogen coexistence. Virology.

[156] Mérai Z., Kerényi Z., Kertész S., Magna M., Lakatos L., Silhavy D. (2006). Double-stranded RNA binding may be a general plant RNA viral strategy to suppress RNA silencing. J. Virol..

[157] Niu S., Wang B., Guo X., Yu J., Wang X., Xu K., Zhai Y., Wang J., Liu Z. (2009). Identification of two RNA silencing suppressors from banana bunchy top virus. Arch. Virol..

[158] Kasschau K.D., Carrington J.C. (2001). Long-distance movement and replication maintenance functions correlate with silencing suppression activity of potyviral HC-Pro. Virology.

[159] Ding X.S., Liu J., Cheng N.-H., Folimonov A., Hou Y.-M., Bao Y., Katagi C., Carter S.A., Nelson R.S. (2004). The Tobacco mosaic virus 126-kDa protein associated with virus replication and movement suppresses RNA silencing. Mol. Plant-Microbe Interact..

[160] Bayne E.H., Rakitina D.V., Morozov S.Y., Baulcombe D.C. (2005). Cell-to-cell movement of potato potexvirus X is dependent on suppression of RNA silencing. Plant J..

[161] Liu J.Z., Blancaflor E.B., Nelson R.S. (2005). The tobacco mosaic virus 126-kilodalton protein, a constituent of the virus replication complex, alone or within the complex aligns with and traffics along microfilaments. Plant Physiol..

